# Microfluidic-Based Amplification-Free Bacterial DNA Detection by Dielectrophoretic Concentration and Fluorescent Resonance Energy Transfer Assisted *in Situ* Hybridization (FRET-ISH) ^†,‡^

**DOI:** 10.3390/bios2040405

**Published:** 2012-10-10

**Authors:** Michelle M. Packard, Maxim Shusteff, Evangelyn C. Alocilja

**Affiliations:** 1 Nanobiosensors Laboratory, Michigan State University, East Lansing, MI 48824, USA; E-Mail: packard2@msu.edu; 2 Lawrence Livermore National Laboratory, 7000 East Ave., Livermore, CA 94550, USA; E-Mail: shusteff1@llnl.gov

**Keywords:** fluorescence resonance energy transfer assisted *in situ* hybridization (FRET-ISH), *in situ* hybridization (ISH), dielectrophoresis (DEP), on-chip diagnostics, lab on a chip, microbial identification

## Abstract

Although real-time PCR (RT-PCR) has become a diagnostic standard for rapid identification of bacterial species, typical methods remain time-intensive due to sample preparation and amplification cycle times. The assay described in this work incorporates on-chip dielectrophoretic capture and concentration of bacterial cells, thermal lysis, cell permeabilization, and nucleic acid denaturation and fluorescence resonance energy transfer assisted *in situ* hybridization (FRET-ISH) species identification. Combining these techniques leverages the benefits of all of them, allowing identification to be accomplished completely on chip less than thirty minutes after receipt of sample, compared to multiple hours required by traditional RT-PCR and its requisite sample preparation.

## 1. Introduction

Prompt public health investigation and response necessitates rapid identification of low bacterial concentrations. This is the case with environmental samples, such as water contamination monitoring, as well as detecting bacterial infections in clinical settings. Although real-time PCR (RT-PCR) is the gold standard for nucleic acid based diagnostics, most PCR protocols remain time-intensive due to sample preparation and amplification cycle times. Presented here is a novel DNA-based diagnostic assay combining dielectrophoretic bacterial capture and concentration, on-chip thermal lysis, cell permeabilization and nucleic acid denaturation with fluorescence resonance energy transfer assisted *in situ* hybridization (FRET-ISH). This approach dramatically reduces the time required for pan-enterobacterial detection to less than thirty minutes from receipt of sample (Table 1), compared to multiple hours needed for traditional RT-PCR and its requisite sample preparation.

**Table 1:** Fluorescence resonance energy transfer assisted *in situ* hybridization (FRET-ISH) assay times.

Bacterial centrifugation and preparation	6 min
Sample delivery to chip	1 min
Dielectrophoretic capture and concentration	1 min
Cell lysis, permeabilization and nucleic acid denaturation	5 min
Nucleic acid hybridization	5 min
Detection and data analysis	5 min
**Total Time**	**23 min**

A DNA probe specific to a region of the enterobacterial repetitive intergenic consensus (ERIC) probe was employed by Torres, *et al*. [1] to detect multiple species of enterobacterial contaminants in environmental slime samples by fluorescent *in situ* hybridization (FISH). That approach was successfully used to detect a range of enterobacteria, specifically in the presence of an environmental matrix, and in the present work that method has been modified to drastically decrease assay time. Whereas the original method achieved detection after a twelve hour incubation period, the reported modification allows detection of probe binding in less than 30 min. 

*Escherichia coli* C3000 was chosen as a representative bacterium for these initial studies; however, the probe itself is designed to detect all enterobacterial species. Therefore this novel method is currently suitable as an initial screening device for early detection. The assay can be refined to more selectively detect particular organisms by choosing species- or strain-specific probes. Reported here is a proof of principle, demonstrating the combination of on-chip operations that enables rapid detection, as well as sufficient speed and assay sensitivity to be used with relevant samples. Further study is required for validation of this approach with more complex sample matrices and other species of bacteria. 

FISH was first introduced in the 1980s and has since found widespread application in bacterial identification [1,2,3,4,5]. Although capable of species-specific microbiological detection, FISH traditionally requires fixation, permeabilization, denaturation, probe hybridization, washing, and detection. Together, the complete process can take greater than twenty-four hours and is often plagued by inadequate sensitivity and specificity [2]. Adaptation of FISH techniques with microfluidic sample preparation steps [6,7,8,9] and fluorescence resonance energy transfer (FRET)-based detection [1] dramatically decreases assay time while increasing both sensitivity and specificity. 

Dielectrophoresis (DEP) offers a fast mechanism for bacterial capture and concentration from small diluted sample volumes. DEP forces arise from the interaction of gradients in non-uniform high frequency (AC) electric fields with dipole moments that are induced in polarizable particles. The sign and magnitude of the forces can be estimated from calculating the real part of the frequency-dependent Clausius-Mossotti factor (Re[F_CM_]), which depends on the relative conductivities and permeabilities of the medium and the particle [10]. In positive dielectrophoresis (pDEP), Re[F_CM_] is greater than 0 and the particle moves up the gradient toward locations of greatest electric field (typically at the edges of electrodes), whereas in negative dielectrophoresis (nDEP), Re[F_CM_] is less than 0 and the particle is repelled from locations of greatest electric field [11]. The device operating frequency is selected to provide the desired DEP regime. The present method imposes fields at approximately 1 MHz to ensure efficient pDEP capture and concentration.

After isolation, cell lysis is a required step for most nucleic acid-based assays [12]. Both off-chip and on-chip methods of lysis have been employed for downstream microfluidic molecular detection of bacteria, including ultrasonic, physical disruption, temperature, and chemical lysis [13,14,15]. Although they are the most common, chemical lysis techniques remain time-consuming and complex due to subsequent purification steps to prevent interference with detectable molecules or assay processes. The present work uses a thermal lysis approach, which uniquely integrates cellular permeabilization and nucleic acid denaturation, and imposes no additional purification requirements.

FRET procedures function on the basic concept of energy transfer between two dyes, a high energy donor and a low energy acceptor, at a certain transfer rate, K_T_ [16]. FRET efficiency (E) is a measure of the donor’s ability to transfer its internal energy to the acceptor, which depends on their physical proximity to each other. In the work reported here, the assay is deliberately designed to have the FRET fluorophores give two different measurements: non-specific labeling of all bacterial DNA (donor) and specific binding to target species (acceptor). The DNA of all intact bacterial cells is first non-specifically stained with SYTO^®^-9, which binds to the minor groove, allowing visualization of all bacteria present. Then, only when a second, enterobacterial-specific 6-Carboxy-2*'*,4,4*'*,5*'*,7,7*'*-hexachlorofluorescein (6-HEX)-labeled probe binds to its complementary DNA sequence will the two fluorophores come within the minimum critical distance necessary for energy transfer. The result is a shift in emission correlated with the number of acceptor-labeled probe binding events, and therefore a measure of the enterobacteria in the population. Use of this method for FRET-ISH detection minimizes the influence of non-specific signals arising from unbound probes that limit traditional FISH assays. 

Probe binding can be detected by measuring emission intensity from the donor or from the acceptor dye. While acceptor emission intensity correlates to probe binding in a straightforward way, measurements from the donor are more complex. The donor fluorophore’s emission signal has an intrinsic decay over time, due to donor photobleaching. When some donor energy transfers to the acceptor due to FRET, the donor’s photobleaching rate decreases, since donor molecules spend less time on average in the excited state [17]. Upon identification of the donor photobleaching rate in the absence of the acceptor, FRET efficiency can then be calculated from the change in decay time due to the presence of an acceptor dye. In order to minimize the complexity of the optics for on-chip experiments, this photobleaching-based method was used for all on-chip detection. Off-chip measurements were carried out at the acceptor’s emission wavelength. 

## 2. Experimental Section

### 2.1. Fluorescent Staining of Cells

*Escherichia coli* C3000 cells (ATCC Cat. No. 15597) were isolated by centrifugation at 5,000 rpm for 5 min at room temperature and resuspended in filtered, distilled water prior to analysis. Then, prior to chip delivery and cell lysis, intracellular bacterial DNA was labeled with Invitrogen^®^ SYTO^®^-9 fluorescent nucleic acid stain (Ex. 488 nm, Em. 500 nm) to monitor dielectrophoretic capture concentration of bacteria [18]. Additionally, the SYTO^®^-9 stain served as the donor dye for the downstream specific FRET-ISH assay. Stained bacteria were then diluted in 10 mL distilled water for a final concentration of 1.23 × 10^6^ cells/mL. This concentration, equivalent to approximately two thousand cells per chip volume, was chosen to ensure significant levels of detection, while preventing crowding during capture. The HEX (Ex. 532 nm, Em. 560 nm) acceptor-tagged enterobacterial repetitive intergenic consensus (ERIC) probe (5′-ATGTAAGCTCCTGGGGATTCAC-3′, T_m_ = 54.8 °C, 11.1 ng/mL) from Integrated DNA Technologies^®^ was added to the bacterial solution immediately prior to dielectrophoretic capture [1]. 

### 2.2. Spectrofluorometry for Confirmation of FRET-ISH Performance

To confirm the performance of the FRET-ISH assay, the method was first performed off chip. Serially diluted bacteria were concentrated by centrifugation at 5,000 rpm for 5 min, resuspended in distilled water, then treated with both SYTO^®^-9 and the ERIC probe. Samples were then heated on a Bio-Rad DNAEngine thermocycler to 65 °C for 5 min for lysis and denaturation followed by incubation at 25 °C to allow probe hybridization. Quantitative analysis of probe signal was then performed using a Nanodrop 3300 Spectrofluorometer (Thermo Scientific) with blue LED excitation at 470 nm. Two microliters of each serially diluted sample were excited at the donor excitation wavelength of 470 nm while acceptor emission at 560 nm was measured to detect energy transfer. 

### 2.3. Dielectrophoretic Capture and Concentration of Cells

**Figure 1:**
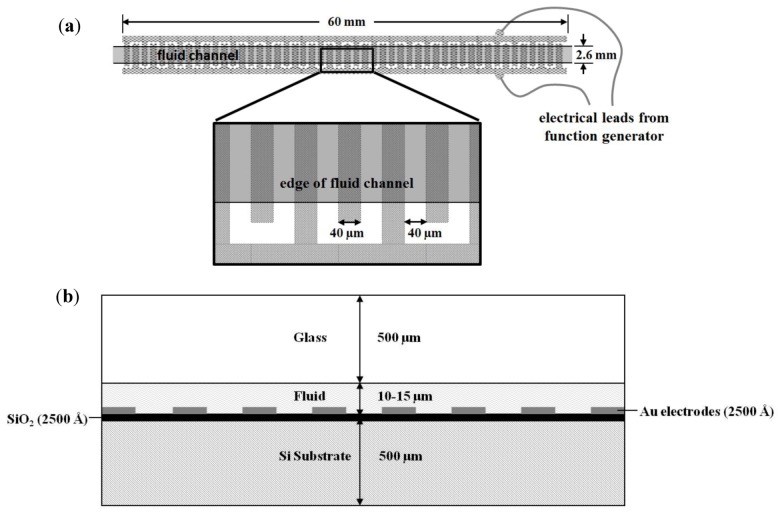
Dielectrophoresis chip design (**a**) top view and (**b**) cross-sectional view. Note that the fluid channel spans only the interdigitated portion of the electrodes, and the metal regions common to each set of electrodes do not come in contact with the fluid.

Dielectrophoresis was performed inside silicon-and-glass chips fabricated using standard cleanroom microfabrication techniques. In brief, a 4*''* silicon wafer was first wet-oxidized to form a 200 nm SiO_2_ insulating layer, on top of which 250 nm of Cr-Au metal was sputter-deposited. The metal was patterned by standard photolithography and wet-etching (AZ 1518 resist, Transene gold etch TFA, Cyantek CR-7 chrome etchant). A second 4*''* wafer made of borosilicate glass was drilled with 500 µm diameter through-holes (Bullen Ultrasonics) to provide fluid access ports. After drilling, a Cr-Au metal layer was sputter-deposited to serve as a mask for fluid channel etching. The fluid channel pattern was wet-etched in the metal mask, and then the glass was etched to a depth of 10–15 µm using a solution of 22% hydrofluoric acid and 78% acetic acid. After stripping the metal etch-mask, the glass and silicon chips were anodically bonded together (350 °C, constant voltage −900 V, ~5 min) to form sealed fluid channels 2.6 mm wide and 60 mm long. Interdigitated electrodes in the chips (Figure 1) were 40 µm wide with 40 µm spacing. Individual chips were diced apart and wire leads were attached using silver paint and epoxy to electrode contact pads connected to each side of the interdigitated electrode array. Serial dilutions of bacteria stained only with SYTO^®^-9 were flowed at 100 µL/min for one minute and dielectrophoretically captured and concentrated at a frequency of 1 MHz and voltage of 10 V_p__-p_ supplied by a standard digital function and waveform generator (Agilent 33220 A) directly to the chip leads. 

### 2.4. On-Chip Probe-Based Identification: FRET-ISH

The mixture of SYTO^®^-9-stained bacteria and HEX-labeled ERIC probe solution in diH_2_O was introduced into the chip by a syringe pump at a fixed rate of 100 µL/min for one minute. As before, cells were dielectrophoretically captured and concentrated at a frequency of 1 MHz and voltage of 10 V_p-p_. 

### 2.5. On-Chip Thermal Lysis, Permeabilization and Nucleic Acid Denaturation and Hybridization

Cells were lysed and permeabilized, and nucleic acids were denatured on-chip at 65 °C for five minutes by a Kapton^®^ KHLV series (Polyimide Film and FEP adhesive) rectangular insulated heater (KHLV-0502/10, 28 V, 1 × 5 cm, 1.6 W/cm^2^) adjacent to the chip and modulated with a thermocouple-attached temperature controller (Alpha Omega Instruments Series 800).

### 2.6. Imaging and Data Analysis

All images were acquired with a ScopeTek DCM200 2.0M pixel CCD camera and MiniSee software. Fluorescent signal was analyzed with ImageJ software to quantify increase in signal from labeled cells indicative of cell concentration and capture [19]. To assess effectiveness of capture and concentration, time-lapse images during bacterial capture by DEP were acquired at a rate of 1 frame/s (100 ms exposure time) for a period of 60 s on a Zeiss Axiovert 5100 filter-based fluorescent microscope. Samples were identically excited with a 485/20 nm filter, and emission from the SYTO-9 donor dye was detected by a 505 nm long-pass filter. FRET-ISH efficiency was determined by time-resolved quantification of donor dye photobleaching. Since the long-pass filter passes both the donor’s and the acceptor’s emission wavelength, images were digitally filtered using the Threshold Colour [19,20] plugin in ImageJ to isolate the green signal (hue values 84–86), prior to quantification of photobleaching. Photobleaching decay time constants were then estimated by exponential fitting for SYTO^®^-9 stained bacterial samples unbound and bound to the HEX-labeled ERIC probe. The FRET efficiency (E) was calculated experimentally as E = 1 − (τ_pb_/τ*'*_pb_) where τ_pb_ = photobleaching decay time constant of the donor without acceptor and τ*'*_pb_ = photobleaching decay time constant of the donor in the presence of acceptor. Quantitative analysis and curve fitting was conducted in Microsoft Excel. All data analysis steps contributed between 60 and 120 s for each photo series.

## 3. Results and Discussion

### 3.1. Spectrofluorometry for Confirmation of FRET-ISH Performance

**Figure 2:**
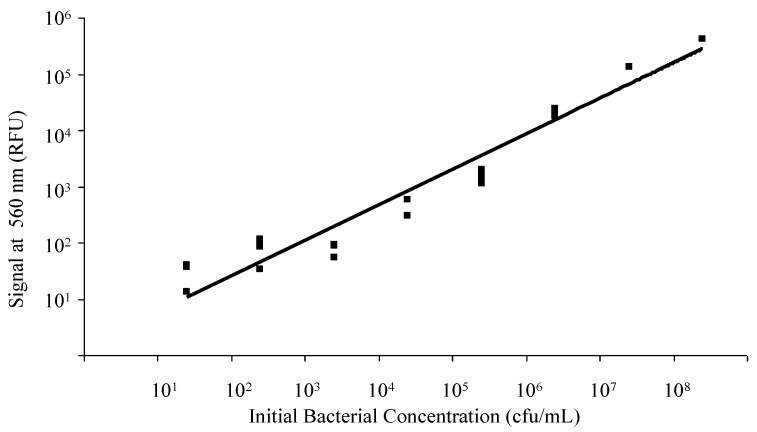
Quantitative Spectrofluorometry. Correlation of bacterial concentration and background subtracted (background = HEX probe signal with no bacterial DNA or donor dye present) FRET-ISH signal (acceptor dye emission at 560 nm) as recorded by the Nanodrop 3300.

Spectrofluorometry on the Nanodrop 3300 allowed quantification of HEX signal and associated labeled probe bound to bacterial DNA previously stained with SYTO^®^-9. Serial dilutions were tested to construct a calibration curve for the relative fluorescence signal intensity as a function of bacterial counts. An increase in HEX (acceptor) emission intensity at 560 nm was detectable for concentrations in the range of 10^1^ to 10^8^ cfu/mL (Figure 2). A small number of other bacterial species which also contain the ERIC sequence in their genome (including *Salmonella enteritidis*, *Klebsiella pneumoniae*, *Streptococcus agalactiae*, and *Escherichia hermanii*) were similarly tested off-chip, showing signal at 560 nm in the presence of the ERIC probe (data not shown).

### 3.2. Dielectrophoretic Capture and Concentration of Cells

**Figure 3:**
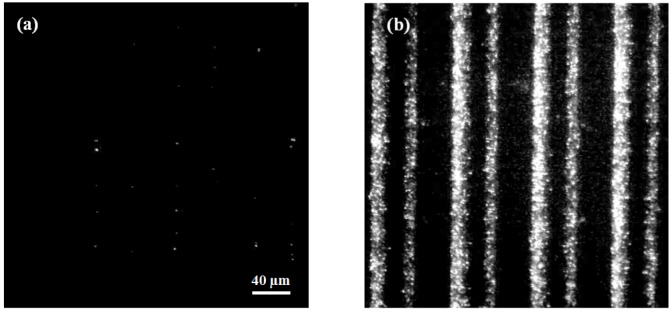
Dielectrophoretic capture and concentration of bacterial cells. Both images have identical camera gain and contrast settings and identical scale/magnification. (**a**) Prior to dielectrophoretic capture and concentration, SYTO^®^-9 stained bacteria (10^6^ cfu/mL) are barely detectable. (**b**) After one minute of capture at 1 MHz and 100 µL/min, bacteria are evident and signal intensity is over 400× greater.

**Figure 4:**
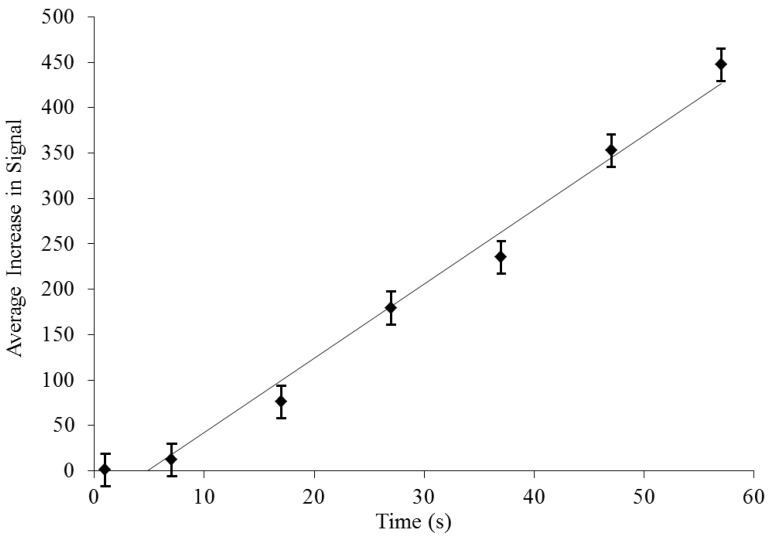
Dielectrophoretic bacterial concentration. While sample solution flows past the electrodes, the total donor fluorescence signal, summed over the entire image, from SYTO^®^-9 labeled bacteria (no acceptor is present) increases linearly over time, measuring more than 400× (458.5 ± 18.2×, n = 6) the initial value after one minute flow at 100 µL/min.

Whereas the initial on-chip cell population is barely detectable at a starting concentration of 10^6^ cfu/mL (Figure 3(a)), bacterial presence at the electrodes after concentration is evident and easily discernible (Figure 3(b)). Flowing at a rate of 100 µL/min for one minute, bacteria were successfully captured and concentrated greater than 400 times by dielectrophoresis (Figure 3 and Figure 4). 

**Figure 5:**
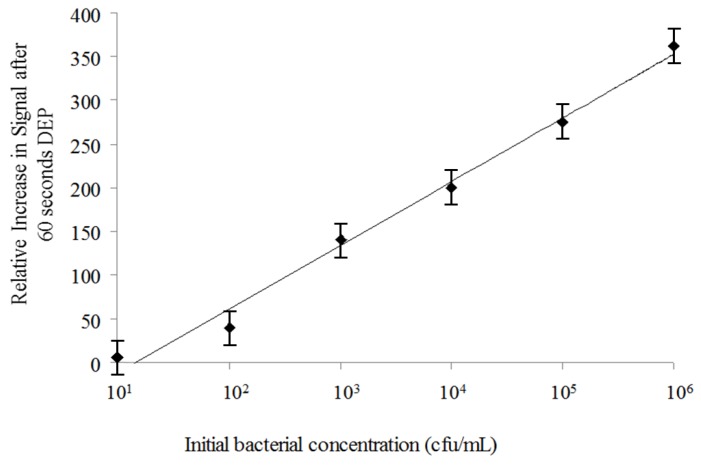
Concentration of serial bacterial dilutions after sixty seconds of dielectrophoresis (DEP). Signal increases with initial bacterial concentration in the range of 10^1^–10^6^ cfu/mL.

Increase in bacterial concentration as measured by SYTO^®^-9 signal was linearly correlated with time (y = 8.0763x − 43.509, R² = 0.9802). One concern with a capture-based approach is that the device might “saturate” or fill with the target particles after a certain time, and fluorescent signal will cease to increase. Because the device did not saturate with captured bacteria at this high starting concentration, we can reasonably expect that it will likewise not saturate at much lower clinically and environmentally relevant bacterial loads and DEP concentration will provide the signal enhancement that enables the sensitivity of this method. Additionally, to confirm detection of bacteria at lower concentrations, a series of measurements were performed on a separate DEP device, successfully concentrating as few as ten cells per mL to a level detectable by SYTO^®^-9 fluorescence (Figure 5).

The observed shift in photobleaching decay time can be used not only to determine the presence or absence of the target species, but to quantify it as well. Provided that the acceptor-labeled probe is present in excess (such that the concentration of bacterial targets defines the number of binding events), the change in decay times correlates with the concentration of target bacteria. Though the present sample set is limited and more detailed investigation will be required to determine the full dynamic range accessible by this technique, the extensive publication record over the last 20 years on quantitative FRET techniques [21,22] indicates that quantitation can be readily achieved. 

### 3.3. On-Chip Probe-Based Identification: FRET-ISH

**Figure 6:**
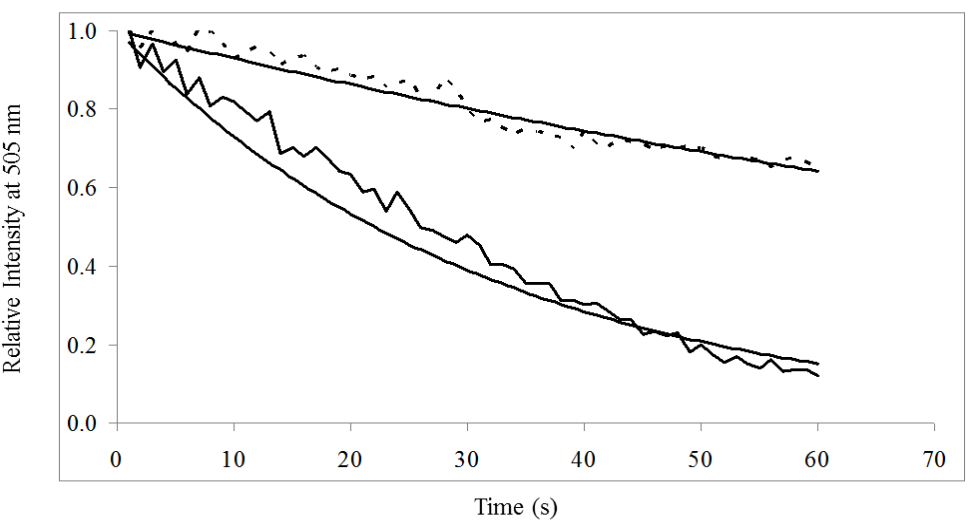
Donor Photobleaching. Photobleaching of donor signal at 505 nm without acceptor (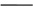) was significantly greater than donor in the presence of acceptor (- - -) when excited at 485/20 nm for 60 s.

FRET-ISH efficiency was determined by quantification of donor dye photobleaching (Figure 6). A decaying exponential fitted to relative intensity over time found photobleaching decay time constant of donor dye alone (τ_pb_) and in the presence of the HEX-labeled ERIC probe (τ*'*_pb_) to be 31.8 s and 135.1 s, respectively (Table 2). Photobleaching decay time constants of emission at 505 nm were calculated for SYTO^®^-9 stained bacterial samples unbound and bound to the HEX-labeled ERIC probe. The FRET efficiency, *E*, was then determined to be 76.4% (Table 2), indicative of exceptional probe binding within nanoscale proximity of the SYTO^®^-9 dye. 

**Table 2:** FRET-ISH photobleaching decay time constants and FRET efficiency.

SYTO^®^-9 alone photobleaching decay time constant (τ_pb_)	31.8 s
SYTO^®^-9 with bound probe photobleaching decay time constant (τ*'*_pb_)	135.1 s
FRET Efficiency (E)	76.4%

## 4. Conclusions

This initial study demonstrates that bringing together the speed and small footprint of on-chip DEP integrated with thermal lysis and in combination with the sensitivity of FRET and the specificity of FISH results in a powerful and compelling new platform for biodetection. This makes the application of the FRET-ISH assay for bacterial detection and identification a promising rapid alternative to traditional RT-PCR. As with other DNA-based tests, FRET-ISH can be easily adapted to the full range of bacteria for which DNA probes are available.

Although photobleaching by filter-based microscopy lacks the precision of laser-driven fluorescence lifetime imaging, the latter method is far more equipment-intensive, and the photobleaching-based approach presented here is far easier to implement and to adapt to a fieldable device. As demonstrated by off-chip spectrofluorometry, integration of a spectrofluorometer to directly measure acceptor emission intensity would allow on-chip quantitation of enterobacteria after dielectrophoretic concentration. It may also be the case that labeling the probe with a donor fluorophore, and using a non-specific acceptor dye may yield better signal quality or a greater dynamic range, but such an alternative design needs to be empirically evaluated. 

As implemented, this novel design is a unique screening tool providing quantitative data of total bacterial populations as well as specific identification of enterobacteria in these populations. Additional validation is necessary to confirm efficacy for real-world samples. In particular, performance with more complex samples in the presence of multiple bacterial species needs to be assessed. Also, the sensitivity limits seen at low concentrations with off-chip measurements must be validated on-chip. For scarce bacterial concentrations, the lower limit of detection can be improved in several ways, such as increasing dielectrophoretic concentration times up to 10-fold without significantly impacting total assay time. In addition, for populations where high capture efficiency is especially important, flow rates can be decreased to maximize percent captured. Future efforts will focus on a more complete characterization of quantitative capabilities of the on-chip method, as well as the performance of the ERIC probe in the presence of non-enterobacterial species. In addition, further research will incorporate probes specific for individual bacterial strains, allowing the choice of more narrowly-defined targets of interest. Overall, by integrating rapid sample concentration and detection with minimal equipment, the FRET-ISH assay shows great potential for future adaptation for field applications.
